# Learning Predictive Interactions Using Information Gain and Bayesian Network Scoring

**DOI:** 10.1371/journal.pone.0143247

**Published:** 2015-12-01

**Authors:** Xia Jiang, Jeremy Jao, Richard Neapolitan

**Affiliations:** 1 Department of Biomedical Informatics, University of Pittsburgh, Pittsburgh, PA 15213, United States of America; 2 Department of Preventive Medicine, Northwestern University Feinberg School of Medicine, Chicago, IL 60611, United States of America; Tampere University of Technology, FINLAND

## Abstract

**Background:**

The problems of correlation and classification are long-standing in the fields of statistics and machine learning, and techniques have been developed to address these problems. We are now in the era of high-dimensional data, which is data that can concern billions of variables. These data present new challenges. In particular, it is difficult to discover predictive variables, when each variable has little marginal effect. An example concerns *Genome-wide Association Studies* (*GWAS*) datasets, which involve millions of *single nucleotide polymorphism* (*SNPs*), where some of the SNPs interact epistatically to affect disease status. Towards determining these interacting SNPs, researchers developed techniques that addressed this specific problem. However, the problem is more general, and so these techniques are applicable to other problems concerning interactions. A difficulty with many of these techniques is that they do not distinguish whether a learned interaction is actually an interaction or whether it involves several variables with strong marginal effects.

**Methodology/Findings:**

We address this problem using information gain and Bayesian network scoring. First, we identify candidate interactions by determining whether together variables provide more information than they do separately. Then we use Bayesian network scoring to see if a candidate interaction really is a likely model. Our strategy is called MBS-IGain. Using 100 simulated datasets and a real GWAS Alzheimer’s dataset, we investigated the performance of MBS-IGain.

**Conclusions/Significance:**

When analyzing the simulated datasets, MBS-IGain substantially out-performed nine previous methods at locating interacting predictors, and at identifying interactions exactly. When analyzing the real Alzheimer’s dataset, we obtained new results and results that substantiated previous findings. We conclude that MBS-IGain is highly effective at finding interactions in high-dimensional datasets. This result is significant because we have increasingly abundant high-dimensional data in many domains, and to learn causes and perform prediction/classification using these data, we often must first identify interactions.

## Introduction

We are in the era of high-dimensional or “big” data. We have data on the gene expression levels of thousands of gene which can be exploited to help provide personalized medical treatments [1]; we have data on millions of single nucleotide polymorphisms which can help us determine the genetic basis of disease [2]; we have abundant passive internet data which can be used for many purposes including learning an individual’s preferences [3] and detecting outbreaks [4]; we have abundant hospital data concerning workflow which can be used to determine good personnel combinations and sequencing [5].

There are several learning tasks involving data. The most straightforward task is simply to look for correlation. For example, we may test a drug versus a placebo, and perform a chi-square test to see if the desired health outcome is correlated with use of the drug. A related task is prediction. For example, we can analyze data on gene expression levels in breast cancer patients not only to see if certain genes are correlated with survival, but also to predict whether a given patient will survive [1]. Going a step further, we can learn a detailed model of the relationships among many variables to develop a rule-based expert systems [6] or a Bayesian network/influence diagram [7]. The model learned can then be used to perform numerous predictions and make decisions. Finally, we can use data to discover causal relationships among variables [8].

Numerous techniques have been developed to perform these learning tasks using low-dimensional data including linear regression and logistic regression, the perceptron [9], support vector machines [10], neural networks [11], and strategies for learning Bayesian network [7]. These techniques do not automatically handle high-dimensional data. However, some have been modified to do so. Techniques include regularized regression and lasso [12], which perform shrinkage. Furthermore, strategies such as ReliefF [13], have been developed to identify possible good predictors in high-dimensional datasets, which can then be provided to another method.

These techniques require that a predictor of a given target has a relatively strong correlation with that target. So, if two or more predictors interact with little marginal effects, the predictors would not be discovered. Table 1 illustrates an interaction with little marginal effects. Variables *X* and *Y* are both trinary predictors of a binary target *Z*. The number next to each variable value shows the fraction of occurrence of that value in the population, and the entries in the table show the probability *Z* equals *z*_1_ given each combination of the predictors. For example,

$$P(z_{1}|x_{1},\;y_{2})=0.11$$

**Table 1:** An interaction with little marginal effect. Variables *X* and *Y* are both trinary predictors of a binary target *Z*. The number next to each variable value shows the fraction of occurrence of that value in the population, and the entries in the table show the probability *Z* equals *z*_1_ given each combination of the predictors.

	*x*_1_ (.25)	*x*_2_ (.5)	*x*_3_ (.25)
*y*_1_ (.25)	0.0	0.1	0.0
*y*_2_ (.5)	0.11	0.0	0.11
*y*_3_ (.25)	0.0	0.1	0.0

We have

$$\begin{array}{l} P(z_{1}|y_{1})=0.0\times 0.25+0.1\times 0.5+0.0\times 0.25=0.05\\ P(z_{1}|y_{2})=0.11\times 0.25+0.0\times 0.5+0.11\times 0.25=0.055\\ P(z_{1}|y_{3})=0.0\times 0.25+0.1\times 0.5+0.0\times 0.25=0.05. \end{array}$$

So although *X* and *Y* together have a strong predictive strength for *Z*, *Y* exhibits little marginal effect. Similarly, *X* also exhibits little marginal effect. We say that *X* and *Y interact* to affect *Z*, where the interaction may or may not be causal.

Epistasis is the interaction of two more genes to affect phenotype. Biologically, epistasis is believed to occur when the effect of one gene is modified by one or more other genes. It is thought that much of genetic risk for disease is due to epistatic interactions with little or no marginal effects [14–17]. The advancement of high-throughput technologies has enabled *Genome Wide Association Studies* (*GWAS*). A *single nucleotide polymorphism* (*SNP*) results when a nucleotide that is typically present at a specific location on the genomic sequence is replaced by another nucleotide. These high dimensional GWAS datasets can concern millions of SNPs, and provide researchers unprecedented opportunities to investigate the complex genetic basis of diseases. By looking at single-locus associations using standard analyses such as chi-squared tests, researchers have identified over 150 risk loci associated with 60 common diseases and traits [18–21]. However, such analyses will miss SNPs that are interacting epistatically with little marginal effect. So, researchers endeavored to develop methods for learning epistasis from high-dimensional GWAS datasets. Traditional techniques such as *logistic regression* (*LR*) [22], *logistic regression with an interaction term* (*LRIT*) [23], penalized logistic regression [24] and Lasso [25] were applied to the task. Other techniques include *multifactor dimensionality reduction* (*MDR*) [26], *full interaction modeling* (*FIM*) [27], using *information gain* (*IG*) alone to investigate only 2-SNP interactions [28], *SNP Harvester* (*SH*) [29], the use of ReliefF [30], random forests [31], predictive rule inference [32], Bayesian *epistasis association mapping* (*BEAM*) [33], *maximum entropy conditional probability modeling* (*MECPM*) [34], and Bayesian network learning [35–37].

The problem of identifying interactions is not limited to SNPs. Predictors could be interacting in all of the situations discussed earlier. So this research on SNP-SNP interactions is applicable to all the problems involving high-dimensional datasets discussed above.

Next, we briefly discuss the *multiple beam search algorithm* (*MBS*), which was developed in [35], to illustrate a difficulty inherent in its methodology. When there are many possible predictors, MBS first uses a Bayesian network scoring criterion (discussed in the Methods Section) to identify the best set of possible predictors. It then initiates a beam from each member of this set. On this beam, it does greedy forward search, adding the predictor that increases the score the most, until no addition increases the score. It then greedily deletes the predictor such that the deletion increases the score the most, until no deletion increases the score. The final set of predictors is a candidate interaction.

There are two problems with this strategy. First, if two predictors each have a strong individual effect, then the model that includes both of them will have a high score and will end up being identified as an interaction, even if they do not interact at all. Second, if several predictors have very strong effects by themselves, they will be chosen on every beam, thereby blocking the beam from finding a lower scoring interacting SNP. The recently developed interaction discovery algorithm REGAL [37] repeatedly runs MBS, each time deleting the SNPs in the highest scoring model. This strategy addresses the second problem, but not the first one.

In this paper we again using MBS to investigate beams, and we use Bayesian network scoring to decide when to stop our search. However, we use information gain to decide which predictor to add instead of adding the predictor that most increases the score. We call the method *MBS-IGain*. We present the results of experiments using 100 simulated datasets comparing MBS-IGain to MBS, REGAL, and 7 other methods.

We note that no method, including ours, can overcome the “curse of dimensionality.” That is, if we have many possible predictors, it is not computationally possible to even investigate every two-predictor combination. We apply MBS-IGain to a real GWAS *late onset Alzheimer's disease* (*LOAD*) data set that concerns data on 312,260 SNPs. In order to obtain computational feasibility, we first determine the 10,000 SNPs with the greatest marginal effect. So, if two SNPs interact with no marginal effect they would likely be missed. Regardless, we obtain interesting results in this real application. Another strategy for pre-processing SNP data is to use to restrict the search space by using knowledge about molecular pathways [38].

## Methods

Our method utilizes Bayesian networks and information gain. So, first we review these two.

### Bayesian Networks

Bayesian networks [6,7,39,40] are increasingly being used for uncertain reasoning and machine learning in many domains including bioinformatics [41–48]. A *Bayesian network* (*BN*) consists of a *directed acyclic graph* (*DAG*) *G* = (*V*, *E*), whose nodeset *V* contains random variables and whose edges *E* represent relationships among the random variables, and a conditional probability distribution of each node *X* ∈ *V* given each combination of values of its parents. Often the DAG is a causal DAG, which is a DAG containing an edge from *X* to *Y* only if *X* is a direct cause of *Y* [7].

**Fig 1:**
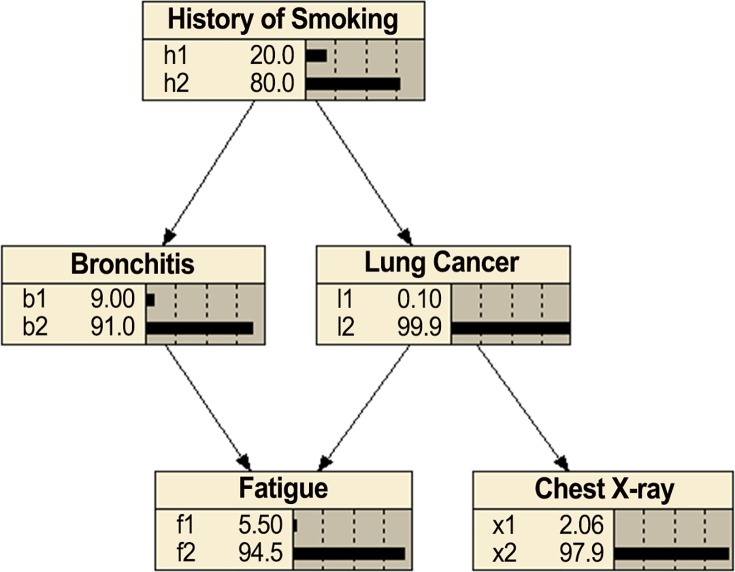
Bayesian network modeling relationships among respiratory diseases.

Fig 1 shows a causal BN modeling the relationships among a small subset of variables related to respiratory diseases (developed using Netica). The values shown at each node are the alternatives and the prior probabilities of the alternatives (times 100). The value *h*1 means the patient has a smoking history and the value *h*2 means the patient does not. The other values have similar meaning.

Using a BN, we can determine conditional probabilities of interest with a BN inference algorithm [7]. For example, using the BN in Fig 1, if a patient has a smoking history (*h*_1_), a positive chest X-ray (*x*_1_), and fatigue (*f*_1_), we can determine the probability of the individual having lung cancer. That is, we can compute *P*(*l*_1_|*h*_1_, *x*_1_, *f*_1_). Algorithms for exact inference in BNs have been developed [7]. However, the problem of doing inference in BNs is NP-hard [49]. So, approximation algorithms are often employed [7].

The task of learning a BN from data concerns learning both the parameters in a BN and the structure (called a DAG model). Specifically, a *DAG model* consists of a DAG *G* = (*V*, *E*) where *V* is a set of random variables, and a parameter set *θ* whose members determine conditional probability distributions for *G*, but without specific numerical assignments to the parameters. The task of learning a unique DAG model from data is called *model selection*. As an example, if we had data on a large number of individuals and the values of the variables in Fig 1, we might be able to learn the DAG in Fig 1 from these data.

In the score-based structure learning approach, we assign a score to a DAG based on how well the DAG fits the data. Cooper and Herskovits [50] developed the Bayesian score, which is the probability of the data given the DAG. This score uses a Dirichlet distribution to represent prior belief for each conditional probability distribution in and contains hyperparameters representing these beliefs. The score is as follows:

$$score_{Bayes}(G\;:\;Data)=P(Data|G)=\prod_{i=1}^{n}\prod_{j=1}^{q_{i}}\frac{\Gamma (\sum_{k=1}^{r_{i}}a_{ijk})}{\Gamma (\sum_{k=1}^{r_{i}}a_{ijk}+\sum_{k=1}^{r_{i}}s_{ijk})}\prod_{k=1}^{r_{i}}\frac{\Gamma (a_{ijk}+s_{ijk})}{\Gamma (a_{ijk})},$$ (1)

where *r*_*i*_ is the number of states of *X*_*i*_, *q*_*i*_ is the number of different instantiations of the parents of *X*_*i*_, *a*_*ijk*_ is the ascertained prior belief concerning the number of times *X*_*i*_ took its *k*th value when the parents of *X*_*i*_ had their *j*th instantiation, and *s*_*ijk*_ is the number of times in the data that *X*_*i*_ took its *k*th value when the parents of *X*_*i*_ had their *j*th instantiation. The parameters *a*_*ijk*_ are known as hyperparameters.

When using the *Bayesian score* we often determine the values of the hyperparameters *a*_*ijk*_ from a single parameter *α* called the *prior equivalent sample size* [51]. If we want to use a prior equivalent sample size *α* and represent a prior uniform distribution for each variable in the network, for all *i*, *j*, and *k* we set *a*_*ijk*_ = *α* / *r*_*i*_ *q*_*i*_. In this case Eq 1 is as follows:

$$score_{\alpha }(G\;:\;Data)=P(Data|G)=\prod_{i=1}^{n}\prod_{j=1}^{q_{i}}\frac{\Gamma (\alpha /q_{i})}{\Gamma (\alpha /q_{i}+\sum_{k=1}^{r_{i}}s_{ijk})}\prod_{k=1}^{r_{i}}\frac{\Gamma (\alpha /r_{i}q_{i}+s_{ijk})}{\Gamma (\alpha /r_{i}q_{i})}.$$ (2)

This version of the score is called the *Bayesian Dirichlet equivalent uniform* (*BDeu*) score.

The *Minimum Description Length (MDL)* score is based on information theory and tries to determine the model that minimizes the number of bits necessary to encode both the model and the data. Suzuki [52] developed the following version of this score:

$$score_{MDL}(G\;:\;Data)=d\frac{{\text{log}}_{2}m}{2}-m\sum_{i=1}^{n}\sum_{j=1}^{q_{i}}\sum_{k=1}^{r_{i}}P(x_{ik},\;pa_{ij})\;{\text{log}}_{2}\frac{P(x_{ik},\;pa_{ij})}{P(x_{ik})P(pa_{ij})},$$ (3)

where *d* is the number of parameters necessary to store the probability distributions, *m* is the number of data items, *r*_*i*_ is the number of states of *X*_*i*_, *x*_*ik*_ is the *k*th state of *X*_*i*_, *q*_*i*_ is the number of instantiations of the parents of *X*_*i*_, *pa*_*ij*_ is the *j*th instantiation of the parents *PA*_*i*_ of *X*_*i*_, and the probabilities are computed using the data.

Note that when we are searching for the best model, we maximize the BDeu score but minimize the MDL score.

To learn a DAG from data we can score all DAGs using the Bayesian or MDL score and then choose the best scoring DAG. However, if the number of variables is not small, the number of candidate DAGs is forbiddingly large. Furthermore, the BN model selection problem has been shown to be NP-hard [53]. So heuristic algorithms have been developed to search over the space of DAGs during learning [7]. These heuristic algorithms assume each parent has a significant marginal effect on the child, and so they cannot learn BNs in which some variables interact with little marginal effect.

### Information Gain

Information theory [54] is the discipline that deals with the quantification and communication of information. If *Z* is a discrete random variable with *m* alternatives, we define the *entropy H*(*Z*) as follows:

$$H(Z)=-\sum_{i=1}^{m}P(z_{i})\;{\text{log}}_{2}P(z_{i}).$$

Shannon [54] showed that if we repeat *n* trials of the experiment having outcome *Z*, then the entropy *H*(*Z*) is the limit as *n* → ∞ of the expected value of the number of bits needed to report the outcome of each trial of the experiment. Entropy is a measure of our uncertainty in the value of *Z* since, as entropy increases, on the average it takes more bits to resolve our uncertainty. Entropy is maximized when *P*(*z*_*i*_) = 1/*m* for all *i*, and is minimized with value 0 when *P*(*z*_*i*_) = 1 for some *i*.

The conditional entropy of *Z* given *X* is the expected value of the entropy of *Z* conditional on *X*. It is defined as follows (where *X* has *k* alternatives):

$$H(Z|X)=\sum_{j=1}^{k}H(Z|x_{j})P(x_{j}),$$

By learning the value of *X*, we can reduce our uncertainty in *Z*. We define the *information gain* of *Z* relative to *X* as the expected reduction in the entropy of *Z* conditional on *X*:

IG(Z;X)=H(Z)−H(Z|X).

The conditional information gain of *Z* relative to *X* conditional on *Y* is the expected value of the information gain conditional on *Y*. It is as follows (where *Y* has *l* alternatives):

$$IG(Z;X|Y)=\sum_{i=1}^{l}IG(Z;X|y_{i})P(y_{i})$$

The following are some important properties of information gain:

1. *IG*(*Z*; *X*) ≥ 0 with equality holding if and only if *Z* and *X* are independent.
2. *IG*(*Z*; *X*) = *IG*(*X*; *Z*).
3. *IG*(*Z*; *X*, *Y*) = *IG*(*Z*; *X* | *Y*) + *IG*(*Z*; *Y*). (chain rule for information gain).
4. *IG*(*Z*; *X*, *Y*) ≥ *IG*(*Z*; *Y*).

The 4th property follows easily from the 1st and 3rd ones.

### Using Information Gain to Measure Interaction Strength

If we have three variables *X*, *Y*, and *Z*, we define the interaction strength of *X* and *Y* relative to *Z* as follows:

IS(Z;X,Y)=IG(Z;X,Y)−IG(Z;X)−IG(Z;Y).

We can generalize this definition to measure interaction strength of *X* and a set of variables A.

IS(Z;X,A)=IG(Z;X,A)−IG(Z;X)−IG(Z;A).

Technically since A is set, we should write {*X*} ∪ A in the *IG* expression. However, we use the more succinct notation. Finally, we can define the interaction strength of the set A as follows:

$$IS(Z;\text{A})=IG(Z;\text{A})-\sum_{X\in \text{A}}IG(Z;X).$$ (4)

Interaction strength measures the increase in information gain obtained by considering *X* and A together relative to considering them separately. We can use the interaction strength to investigate interactions. The greater its value, the more indication we have that *X* and the variables in A are interacting to affect *Z*. In this way, we can discover variables that have little marginal effect while have a large effect together. Furthermore, we should be less likely to conclude that two non-interacting variables with strong individual effects are interacting because their individual information gains will be high, while their joint gain should not add much. In this next section we develop an algorithm for learning interactions that uses the interaction strength.

**Fig 2:**
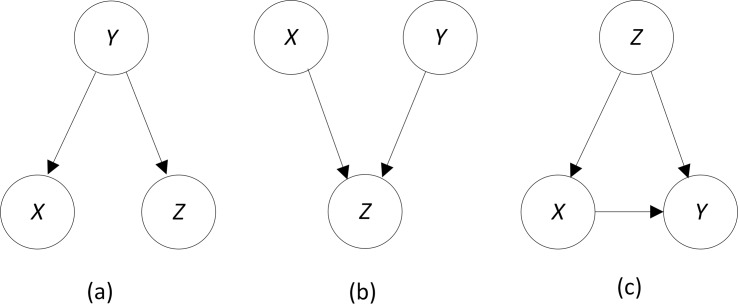
Causal BN DAG models.

In general, the interaction strength can be positive or negative. Fig 2 shows three causal BN DAG models illustrating some of the possibilities. In Fig 2(A), *X* and *Z* are independent conditional on *Y*. We then have by Properties (1) and (3) above

$$\begin{array}{l} IG(Z;X,Y)=IG(Z;X|Y)+IG(Z;Y)\\ \;=0+IG(Z;Y)=IG(Z;Y). \end{array}$$

We therefore have by Property (1) above

$$\begin{array}{l} IS(Z;X,Y)=IG(Z;X,Y)-IG(Z;X)-IG(Z;Y)\\ \;=IG(Z;Y)-IG(Z;X)-IG(Z;Y)\\ \;=-IG(Z;X)\leq 0. \end{array}$$

On the other hand, in Fig 2(B) *X* and *Y* are independent causes of *Z*. If *X* and *Y* are independent, we have the following by Properties (1) and (3) above

$$\begin{array}{l} IG(X;Z|Y)=IG(X;Z,Y)-IG(X;Y)\\ \;=IG(X;Z,Y). \end{array}$$

So, by Properties (2), (3) and (4)

$$\begin{array}{l} IS(Z;X,Y)=IG(Z;X,Y)-IG(Z;X)-IG(Z;Y)\\ \;=IG(Z;X|Y)+IG(Z;Y)-IG(Z;X)-IG(Z;Y)\\ \;=IG(X;Z,Y)-IG(Z;X)\\ \;=IG(X;Z,Y)-IG(X;Z)\\ \;\geq 0. \end{array}$$

In many applications the variables that we are investigating for interactions are known to be independent possible causes. For example, in a GWAS data set, the causal SNPs are often independent possible causes of the disease. In these cases we can be assured that the interaction strength is non-negative.

We stress that, in general, a high value of the interaction strength does not imply that the investigated variables are causes of the target. Consider the causal DAG model in Fig 2(C). Since this complete DAG can represent any joint probability distribution of three variables, we could have the same interaction strength with this underlying causal mechanism as we would have when *X* and *Y* have a strong interactive causal effect on *Z*. So, in an agnostic search for interactions, we cannot assume that a discovered interaction is causal. For example, if we are searching for interactions when developing a BN DAG model and make every variable a target, we cannot assume that what appears to be a discovered interaction is a causal interaction.

### Information Gain and the MDL Score

Suppose we have the simple DAG model in which there is a target *Z* with parent set *PA*. Then the local MDL score at node *Z* is as follows:

$$score_{MDL}(Z;PA\;:\;Data)=d\frac{{\text{log}}_{2}m}{2}-m\sum_{j=1}^{q}\sum_{k=1}^{r}P(z_{k},\;pa_{j})\;{\text{log}}_{2}\frac{P(z_{k},\;pa_{j})}{P(z_{k})P(pa_{j})},$$

where *d* is the number of parameters necessary to store the conditional distributions for *Z*, *m* is the number of data items, *r* is the number of states of *Z*, and *q* is the number of values the parents of *Z* can take. By *score*_*MDL*_ (*Z*; *PA*: *Data*) we mean the MDL score of the model that has the variables in *PA* as the parents of *Z* and no other edges.

It is possible to show that

$$\sum_{j=1}^{q}\sum_{k=1}^{r}P(z_{k},\;pa_{j})\;{\text{log}}_{2}\frac{P(z_{k},\;pa_{j})}{P(z_{k})P(pa_{j})}=IG(Z;PA).$$

Note that in this context the *IG* also depends on *Data* since we use the data to compute the probabilities. However, we do not show that dependency. So, the MDL score is given by

$$score_{MDL}(Z;PA\;:\;Data)=d\frac{{\text{log}}_{2}m}{2}-m\times IG(Z;PA),$$

Recall that smaller scores are better. So, larger information gain improves the score.

### Algorithm for Discovering Interactions

We assume that we have possible predictors *X*_*i*_ and a target variable *Z*. We want to find predictors that interact to affect *Z*. The algorithm that follows does this.

Algorithm *Jiang-MBS-IGain*

Determine the set *Best* of *n* highest scoring predictors *X*_*i*_ using *score*(*Z*;*X*_*i*_);

for each predictor *X*_*i*_ ∈ *Best*

*G*_*i*_ = {*X*_*i*_};

*flag* = 0;

while *flag* = 0

Determine predictor *X* that maximizes *IS*(*Z*;*G*_*i*_, *X*);

if

$$\frac{IS(Z;G_{i},\;X)}{IG(Z;G_{i})+IG(Z;X)}\leq T\;\text{or}\;score(Z;G_{i},X)<score(Z;G_{i})$$

*flag* = 1;

else

add *X* to *G*_*i*_;

endelse

endwhile

endfor

**Fig 3:**
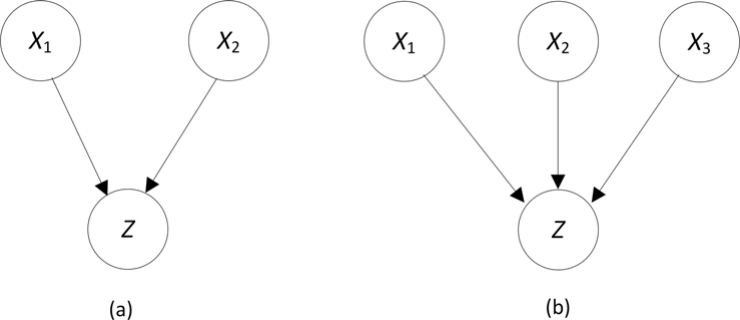
If the DAG model in (b) has a lower score than the one in (a), we do not add node *X*_3_.

Sort the *n* models by *score*(*Z*;*G*_*i*_);

Output the sorted list;

The *score*(Z;*G*_*i*_) in Algorithm *MBS-IGain* is either the MDL score or the BDeu score of the BN DAG model that has the variables in *G*_*i*_ as parents of our target variable *Z*. By *score*(*Z*:*X*_*i*_) we mean only *X*_*i*_ is the parent of *Z*. This algorithm synergistically use Bayesian network scoring criteria and the *IS* and *IG* functions. First, if there are too many predictors to investigate all of them, the most promising predictors are selected using the scoring criterion. A beam is then started from each of these predictors. On each beam, we greedily select the predictor that has the highest *IS* with the set of predictors chosen so far. We end the search when either the *IS* is small relative to the individual *IG*s (as determined by a threshold *T*), or when adding the predictor decreases the score of the model. This latter exit criterion is important because we not only want to discover predictors that appear to be interacting, but we also want to discover likely models. Fig 3 illustrates the situation. Suppose our current model *G*_*i*_ is the one in Fig 3(A), and we find that *X*_3_ has the greatest interaction strength with variables already in the model, and the interaction is sufficiently strong that we exceed the threshold. If the model in Fig 3(B) has a lower score than the one in Fig 3(A), then it is less likely that all three nodes are parents of *Z*, and so we do not add *X*_3_ in spite of the interaction strength being relatively large. On the other hand, the check for a sufficiently large *IS* is important because two or more SNPs could score very high as parents of *Z* when there is no evidence of an interaction. An example would be when *X* and *Y* each have strong causal strengths for *Z* but affect *Z* independently, as specified in the Noisy-OR model [3,7]. In this case the model *X*→*Z*←*Y* has a high score, even though there is no interaction.

Let *n* be the number of predictors investigated. There are *n* passes through the for-loop in MBS-Gain. In the worst case, there are *n*(*n*-1)/2 passes through the while-loop for each pass through the for-loop. So, technically the algorithm is *O*(*n* ^3^). However, we stop developing each model after *M* predictors are added, where *M* is a parameter. In our experiments, *M* = 4. So in practice the algorithm is *O*(*n* ^2^).

We noted previously that the MDL score can be expressed in terms of *IG* as follows:

$$score_{MDL}(Z;PA\;:\;Data)=d\frac{{\text{log}}_{2}m}{2}-m\times IG(Z;PA),$$

Using this equality and some algebraic manipulation, it is possible to show the following (We do not show the dependence on *Data* for the sake of brevity):

$$IS(Z;X,\text{A})=\left(score_{MDL}(Z;X)\;+score_{MDL}\;(Z;\text{A})-score_{MDL}(Z;X,\text{A})\right)/m+C$$

where *C* is a constant. Since *C* and *m* are the same for all predictors *X*, when doing our search in Algorithm *MBS-IGain*, we could just find the predictor that maximizes the following expression:

$$score_{MDL}(Z;X)+score_{MDL}(Z;\text{A})-score_{MDL}(Z;X,\text{A}).$$

This result motivates investigating the use of the BDeu score in our search instead of the MDL score. That is, we find the predictor *X* that maximizes this expression:

$$\text{ln}\;score_{\alpha }(Z;X,\;A)-\text{ln}\;score_{\alpha }(Z;X)-\text{ln}\;score_{\alpha }(Z;A).$$

Jiang et al. [55] had better results discovering interactions using the BDeu score than using the MDL score. So, we might obtain improved results by using the BDeu score in our interaction strength formula. However, when we tried doing this in our studies, our results were worse than those obtained using the true *IS*. So, we do not include this strategy in the experimental results that follow.

### Experiments

Chen et al. [56] generated datasets based on two 2-SNP interactions, two 3-SNP interactions, and one 5-SNP interaction, making a total of 15 causative SNPs. The effects of the interactions were combined using a Noisy-OR model [3,7]. The specific interacting SNPs were as follows:

1. {S1, S2, S3, S4, S5}
2. {S6, S7, S8}
3. {S9, S10, S11}
4. {S12, S13}
5. {S14, S15}

Three parameters were varied to create the interactions: 1) *θ*, which determined the penetrance; 2) *β*, which determined the minor allele frequency; and *l*, which determined the linkage disequilibrium of the true causative SNPs with the observed SNPs. See [56] for details concerning these parameters. For various combinations of these parameters, Chen et al. [56] developed datasets containing 1000 cases and 1000 controls. In our evaluation, we used the 100 1000-SNP datasets they developed with *θ* = 1, *β* = 1, and *l* = *null*.

We compared the performance of MBS-IGain, MBS [35], and REGAL [37] using these 100 datasets. MBS is similar to MBS-IGain except that in the forward search it adds the predictor that increases the score rather than the interaction strength the most. It also does backward search deleting the predictor that increases the score the most. REGAL repeatedly runs MBS, each time deleting the predictors in the highest scoring interaction. By eliminates the predictors in the highest scoring interaction, we guarantee that in the next iteration they won’t be chosen on any of the beams. In this way, lower scoring interacting predictors can be found in the subsequent iteration.

We ran all three methods with the MDL score and the BDeu score with α = 1, 4, 9, 54, and 128. For MBS-IGain we used threshold values of *T* = 0.01, 0.05. 0.1, 0.15, 0.2, 0.3, 0.4, 0.5. We configured REGAL to repeatedly run MBS 5 times. For all three methods, we limited the number of SNPs in a model to 5, which means at most *M* = 4 SNPs were added on each beam.

The experiments were run on three computers. JLJ69-dt contains an Intel i7 4790k with 32GB of memory which runs on Linux Mint 17.1 and Oracle Java 8 64-bit. The two others, DBGAP and DBGAP2, contain identical two AMD Opteron 4280s per computer with 128GB of memory, 64GB per NUMA node, which run on Windows Server 2008 R2 and Oracle Java 8 64-bit. Each experiment involving the 100 datasets was run using a shell script to run a java-runtime file which iterated on the datasets in question, with each parameter on its own thread spread among the three computers. The script was run the same for each of the configurations mentioned above.

We compared the methods using the following two criteria:

#### Criterion 1

This criterion determines how well the interacting predictors (SNPs), are discovered without regard to whether the actual interactions themselves are discovered. It measures the frequency with which the interacting predictors are ranked among the first *K* predictors. First, the learned interactions are ordered by their scores. Then the predictors are ordered according to the first interaction in which each appears. Finally, the power according to criterion 1 is computed as follows:

$$Power_{1}(K)=\frac{1}{R\times M}\sum_{i=1}^{R}N_{K}(i)$$

where *N*_*K*_(*i*) is the number of interacting predictors appearing in the first *K* predictors for the *i*th dataset, *M* is the total number of interacting predictors, and *R* is the number of datasets. In our comparison experiments using the 100 1000 SNP datasets *M* = 15 and *R* = 100.

#### Criterion 2

This criterion measures how well each of the given interactions is discovered. Each interaction is investigated separately. The power is computed using the Jaccard index which is as follows:

$$Jaccard(A,B)=\frac{\#(A\cap B)}{\#(A\cup B)}.$$

This index is 1 if the two sets are identical and is 0 if they have no items in common. First, the learned interactions are ordered by their scores for each dataset *i*. Let *M*_*j*_ (*i*) denote the *j*th learned interaction in the *i*th dataset, and *C* denote the true interaction we are investigating. For each *i* and *j* we compute *Jaccard*(*M*_*j*_(*i*), *C*). Then we set

$$J_{K}(i,C)=\underset{1\leq j\leq K}{\text{max}}\;Jaccard(M_{j}(i),C)$$

Then the power according to criterion 2 is computed as follows:

$$Power_{2}(K,C)=\frac{1}{R\times M}\sum_{i=1}^{R}J_{K}(i,C)$$

where *M* is the total number of interacting predictors, and *R* is the number of datasets.

Reiman et al. [57] developed a GWAS *late onset Alzheimer's disease* (*LOAD*) data set that concerns data on 312,260 SNPs and contained records on 859 cases and 552 controls. We applied MBS-IGain to this dataset to investigate how well it can learn interactions in a real setting. In order to apply MBS-IGain to this dataset, it was necessary to first filter the SNPs because 312,260 is too many SNPs to handle. One of the 312,260 loci is the APOE gene; however, we still use the terminology “SNP” to refer to the loci. We filtered by choosing the 1000 top-scoring 1-SNP models, the 5000 top-scoring 1-SNP models, and the 10,000 top-scoring 1-SNP models, and ran MBS-IGain with each of these sets of SNPs. We also ran the top-scoring 100 SNP models with all the remaining SNPs. We used the BDeu Score with α = 4, the MDL score, and thresholds of 0.05 and 0.1. We limited the size of the models to 5, which means at most M = 4 SNPs were added on each beam.

### Ethics Statement

This research uses only simulated datasets and a real de-identified dataset. So it does not require IRB approval.

## Results

### Simulated Datasets

First, we compare the result of using MBS-IGain with the MDL score to its results using the BDeu score. For values of the threshold that are not extreme (0.05 to 2), the results were the best and were very similar. Furthermore for smaller values of α in the BDeu score (1, 4, 9), the results were the best and were very similar. We show the results that were obtained with T = 0.1 for both methods and with α = 4 for the BDeu score. S1 Fig shows *Power*_1_(*K*) for *K* ≤ 100 for the two scores. S2 Fig shows *Power*_2_(*K*,*C*) for *K* ≤ 100 for each of the 5 interactions for the two scores; S2 Fig (f) shows the average of *Power*_2_(*K*,*C*) over all 5 interactions. We see that the BDeu score sometimes performed slightly better and the MDL score sometimes performed slightly better. Overall, the two scores yield very comparable results.

**Fig 4:**
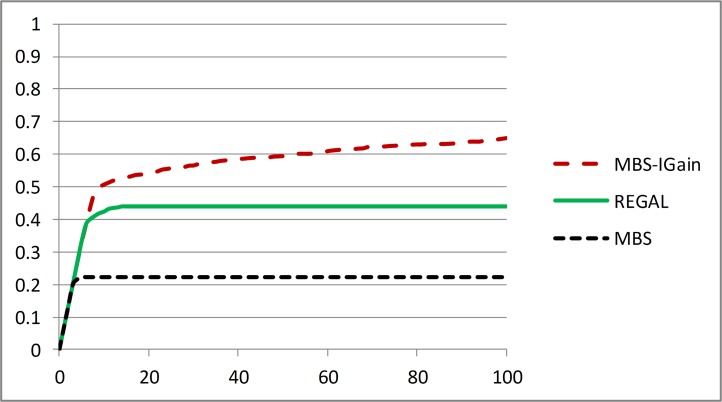
Comparison of MBS-IGain, REGAL, and MBS all using the MDL score (with *T* = 0.1 for MBS-IGain) according to performance Criterion 1.

**Fig 5:**
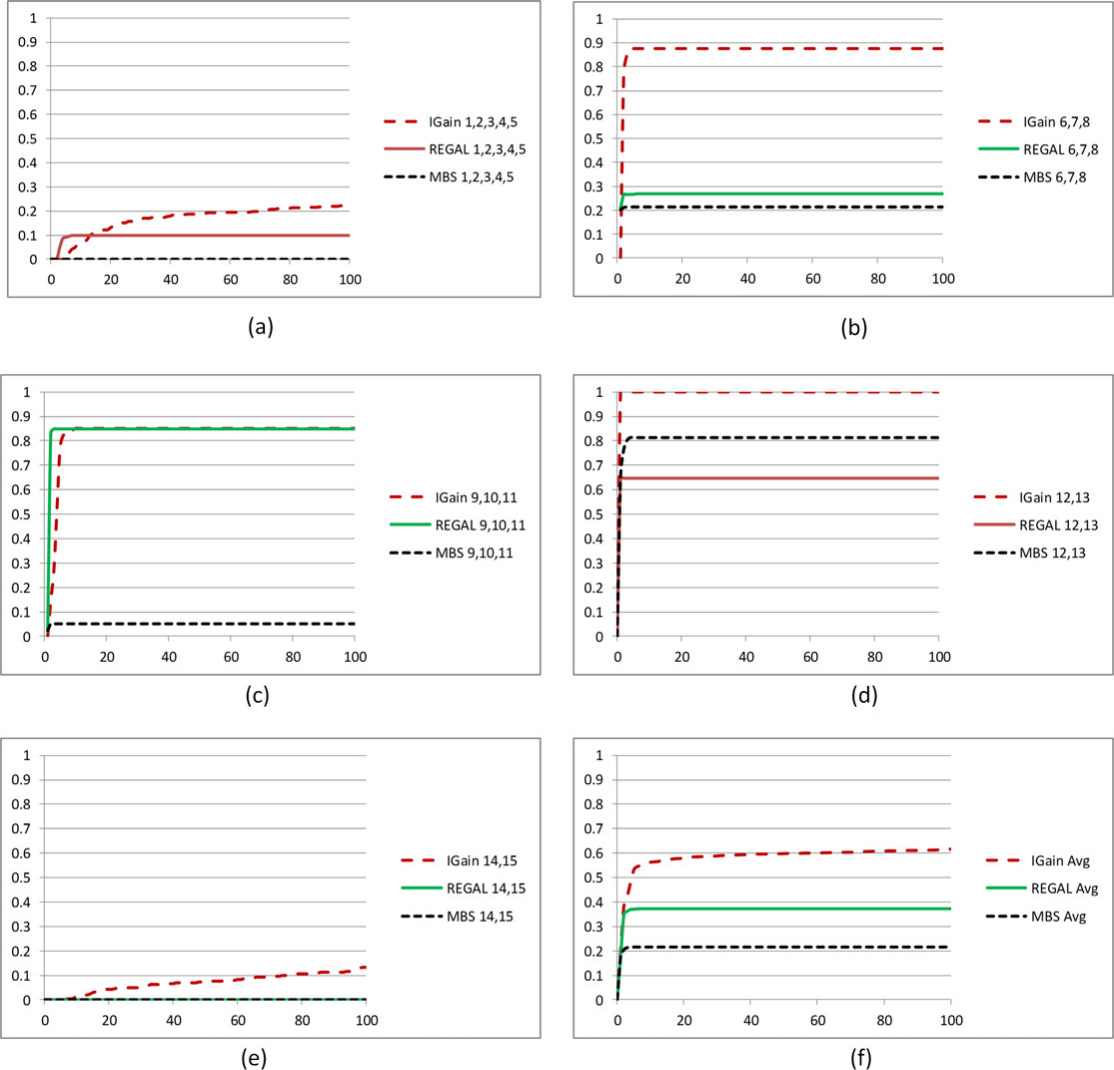
Comparison of MBS-IGain, REGAL, and MBS all using the MDL score (with *T* = 0.1 for MBS-IGain) according to performance Criterion 2.

Next, we compare the results for MBS-IGain, REGAL, and MBS. Like MBS-IGain, the performances of REGAL and MBS were best when using the MDL score or the BDeu score with smaller values of α. The results we show are for when all three methods use the MDL score and MBS-IGain uses a threshold of *T* = 0.1. Fig 4 shows *Power*_1_(*K*) for *K* ≤ 100 for the three methods. Fig 5 shows *Power*_2_(*K*,*C*) for *K* ≤ 100 for each of the 5 interactions *C* for the three methods; Fig 5(F) shows the average of *Power*_2_(*K*,*C*) over all 5 interactions. Looking at Fig 4 and Fig 5(F) we see that REGAL performs notably better than MBS both at locating the interacting SNPs and at identifying the interactions exactly. So, the strategy of removing interactions and repeatedly running MBS seems to pay off. However, MBS-IGain performs notably better than REGAL at both these tasks. So, it appears that a much better strategy than repeatedly running MBS is to use information gain to identify interactions and then using BN scoring to decide whether the learned interaction is a likely model. MBS-IGain locates over half of the interacting SNPs within the first 10 predictors identified, and locates almost 2/3 of them in the first 100 predictors.

We now discuss the results for each interaction individually to illustrate the advantage of using information gain and BN scoring instead of using BN scoring alone. We look at the interactions in the order in which MBS-IGain is capable of identifying them. Fig 5(D) shows that MBS-IGain always locates interaction {S12,S13} exactly and it is the highest scoring interaction. That is, the average Jaccard Index for this interaction jumps to 1 at *K* = 1. Both REGAL and MBS are fairly good at locating this interaction early, but their Jaccard Indices are smaller (MBS actually performs better than REGAL for this interaction). The problem is that they include extra SNPs (ordinarily S6) in the model because the extra SNP increases the score. MBS-IGain does not add this extra SNP because it has weak interaction strength with {S12,S13}. Fig 5(B) shows that MBS-IGain is good at locating interaction {S6,S7,S8} early, but REGAL and MBS are not. The problem is that they usually group S6 with {S12,S13}, and then often do not find S7 and S8 at all. We see from Fig 5(C) that both MBS-IGain and REGAL are good at locating interaction {S9,S10,S11} early but MBS is not. According to that figure REGAL outperforms MBS-IGain slightly. However, this is a little misleading because MBS-IGain always ranks {S12,S13} first and often ranks {S6,S7,S8} second. REGAL does not do this as often. So it identifies {S9,S10,S11} slightly earlier. Fig 5(A) reveals none of the methods are very good at identifying {S1,S2,S3,S4,S5}; however, overall MBS-IGain does best. Again, REGAL does slightly better early. We see from Fig 5(E) that both REGAL and MBS cannot identify {S14,S15} at all, whereas MBS-IGain identifies it to a limited extent. This interaction is particularly difficult to learn because the penetrance for the interacting SNPs is only 0.07. By way of contrast, the penetrances for interaction {S12,S13} are 0.5 and 0.7. See [46] for the details of the 5 models.

S1 Table shows the average running times for the three methods when analyzing the 100 1000 SNP datasets. Not only did MBS-IGain perform substantially better than the other methods, but it also was the fastest. This result is apparent based on a simple analysis of the algorithms. MBS-IGain only does a forward search, whereas MBS does forward and backward search, and REGAL repeatedly runs MBS (in our experiments 5 times). Since the algorithms are quadratic-time, an extrapolation of the results for the MDL score in S1 Table indicates that it would take MBS-IGain about 12 days to handle 20,000 predictors, while it would take REGAL around two months. This time difference could be significant in a real search. The computation of the BDeu score appears to be faster than that of the MDL score based on S1 Table.

**Fig 6:**
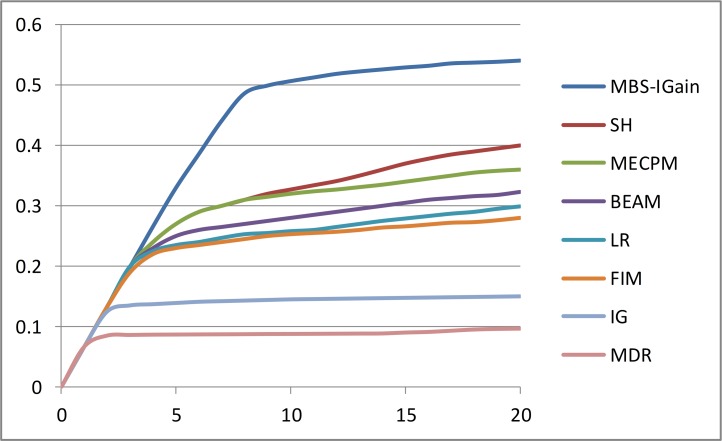
Comparison of MBS-IGain to 7 other methods according to performance Criterion 1. SNP Harvester (SH), maximum entropy conditional probability modeling (MECPM), Bayesian epistasis association mapping (BEAM), logistic regression (LR), full interaction modeling (FIM), information gain (IG), multifactor dimensionality reduction (MDR).

Fig 6 shows a comparison of MBS-IGain to 7 other methods according to performance Criterion 1. The 7 methods are as follows: *logistic regression* (*LR*) [22], *multifactor dimensionality reduction* (*MDR*) [26], *full interaction modeling* (*FIM*) [27], *information gain* (*IG*) [28], *SNP Harvester* (*SH*) [29], Bayesian *epistasis association mapping* (*BEAM*) [33], and *maximum entropy conditional probability modeling* (*MECPM*) [34]. The same 100 1000 SNP datasets were used in the comparison. That is, they are the datasets developed by Chen et al. [56] with *θ* = 1, *β* = 1, and *l* = *null*. The results for the other 7 methods were obtained from Chen et al. [56]. As we see from Fig 6, MBS-IGain exhibited substantially better performance than the other 7 methods according to performance Criterion 1.

**Fig 7:**
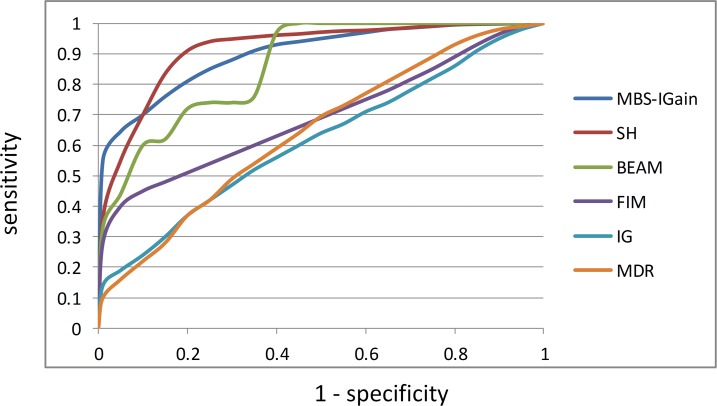
ROC curves for MBS-IGain and 5 other methods. SNP Harvester (SH), Bayesian epistasis association mapping (BEAM), full interaction modeling (FIM), information gain (IG), multifactor dimensionality reduction (MDR).

Six of the 8 methods produce a total ranking of all the SNPs while learning interactions. For these methods we can develop ROC curves. To obtain one point on the ROC curve, we determine the number *x* of true SNPs (ones involved in interactions) and the number *y* of false SNPs in the top *m* SNPs. Based on this value of *m*, the true positive rate (sensitivity) is *x*/15 and the false positive rate (1-specificity) is *y*/985. We repeat this calculation for many values of *m* spaced between 0 and 1000. Fig 7 shows the ROC curves for the 6 methods. MBS-Gain has a true positive rate of about 0.5 with a negligible false positive rate. SH has only around a 0.4 true positive rate with a negligible false positive rate. However, by the time we get to a false positive rate of 0.1, SH overtakes MBS-IGain, and SH is in turn overtaken by BEAM at a false positive rate of 0.4. So, if we can tolerate a 10% false positive rate, LH might be a better choice. However, ordinarily we want to keep the false positive rate much smaller than 10%.

### Real Datasets

**Fig 8:**
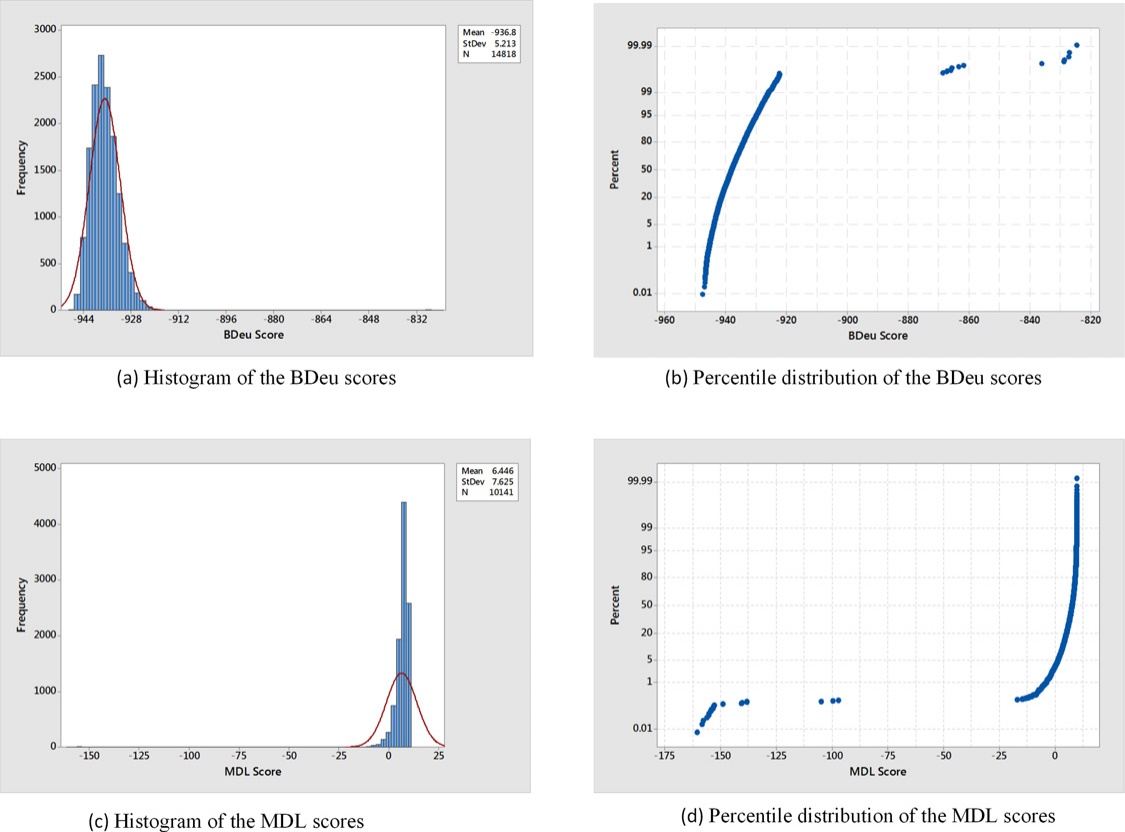
Histograms and percentile distributions when determining interactive models from the LOAD dataset.

When analyzing the real LOAD GWAS dataset with MBS-IGain, the BDeu score discovered a total of 14,818 unique models and the MDL score discovered a total of 10,141 unique models. Fig 8A shows a histogram of the BDeu scores and Fig 8B shows the percentile distribution. We see that the vast majority of the BDeu score approximately follow a normal distribution; however, there are 14 outliers with much higher scores. Fig 8C shows a histogram of the MDL scores and Fig 8D shows the percentile distribution. Although the distribution of the MDL scores is not a close to being normal as that of the BDeu score, most of the scores are clumped together and there are 21 outliers. The outliers for both scores constitute notable findings.

Table 2 shows the notable interactions discovered by the BDeu score, and Table 3 shows the notable interactions discovered by the MDL score. For both scores the notable finding distribute into two groups. The first group contains higher-scoring interactions involving the APOE gene, and the second group contains lower-scoring interactions involving the APOC1 gene. APOE is the strongest genetic predictor of LOAD. Furthermore APOE and APOC1 are in linkage disequilibrium, and APOC1 predicts LOAD almost as well as APOE [58]. Our results indicate that every notable interaction includes one of them.

**Table 2:** Interactions learned from the LOAD dataset using the BDeu score with α = 4. The third column shows gene on which the SNP resides if it is located in a gene; otherwise it shows the chromosome. The fourth column shows the BDeu score of the interaction, and the fifth column shows the interaction strength of the interaction (See Eq 4).

Rank	Interaction	Genes	BDeu	IS
1	rs10510511, rs197899, rs7115850, APOE	Chrome 3, Chrome 6, GAB2, APOE	-824.6	0.042
2	rs11895074, rs7115850, APOE	SPAG16, GAB2, APOE	-827.1	0.035
3	rs536128, rs7115850, APOE	CALN1, GAB2, APOE	-827.3	0.020
4	rs7101429, rs10510511, rs197899, APOE	GAB2, Chrome 3, Chrome 6, APOE	-828.7	0.039
5	rs11122116, rs16856748, rs734600, rs16992170, APOE	NPHP4, LRP2, EYA2, EYA2, APOE	-828.9	0.049
6	APOE	APOE	-836.3	0
7	rs41369150, rs2265264, rs11217838, rs4420638	FNDC3B, Chrome 10, ARHGEF12, APOC1	-861.8	0.049
8	rs7355646, rs41369150, rs4420638	Chrome 2, FNDC3B, APOC1	-863.4	0.023
9	rs7355646, rs41528844, rs4420638	Chrome 2, ADAMTS16, APOC1	-865.6	0.018
10	rs2265264, rs4420638	Chrome 10, APOC1	-865.9	0.026
11	rs41369150, rs4420638, rs6121360	FNDC3B, APOC1, TM9SF4	-866.1	0.019
12	rs10922885, rs7355646, rs4420638	Chrome 1, Chrome 2, APOC1	-867.3	0.023
13	rs41369150, rs4420638	FNDC3B, APOC1	-867.4	0.010
14	rs7355646, rs4420638	Chrome 2, APOC1	-868.6	0.012

**Table 3:** Interactions learned from the LOAD dataset using the MDL score. The third column shows the gene on which the SNP resides if it is located in a gene; otherwise it shows the chromosome. The fourth column shows the negative MDL score of the interaction, and the fifth column shows the interaction strength of the interaction (See Eq 4).

Rank	Interaction	Genes	MDL	IS
1	rs7115850, APOE	GAB2, APOE	160.5	0.013
2	rs197899, APOE	Chrome 6, APOE	158.3	0.009
3	rs1785928, APOE	ELP2, APOE	157.6	0.008
4	rs10793294, APOE	GAB2, APOE	156.0	0.012
5	rs41491045, APOE	Chrome 2, APOE	155.3	0.009
6	rs2057537, APOE	TCP11, APOE	155.2	0.007
7	APOE	APOE	154.7	0
8	rs12421071, APOE	Chrome 11, APOE	154.2	0.007
9	rs891159, APOE	ATG10, APOE	154.2	0.009
10	rs12674799, APOE	Chrome 8, APOE	153.4	0.010
11	rs1957731, APOE	Chrome 4, APOE	153.3	0.009
12	rs1389421, APOE	Chrome 11, APOE	153.0	0.007
13	rs986647, APOE	Chrome 4, APOE	153.0	0.009
14	rs17095891, rs8108841, APOE	Chrome 10, Chrome 19, APOE	152.8	0.016
15	rs2717389, rs10130967, APOE	Chrome 3, Chrome 14, APOE	140.7	0.022
16	rs898717, rs16975605, APOE	FRMD4A, Chrome 15, APOE	140.5	0.020
17	rs11895074, rs7115850, APOE	SPAG16, GAB 2, APOE	138.2	0.035
18	rs11676052, rs6719419, APOE	Chrome 2, Chrome 2, APOE	138.1	0.020
19	rs2265264, rs4420638	Chrome 10, APOC1	104.9	0.026
20	rs41369150, rs4420638	FNDC3B, APOC1	99.7	0.010
21	rs4420638	APOC1	97.1	0

All the notable findings can provide LOAD researchers with candidate interactions which they can investigate further. We discuss some of the more interesting ones. The MDL score, with its larger DAG penalty, discovers two 2-locus models involving the GAB2 gene. A good deal of previous research has indicated that GAB2 and APOE interact to affect LOAD [57]. The MDL score also discovers several other 2-locus models containing APOE. The BDeu score, with its smaller DAG penalty, discovers as its highest scoring interaction, a 4-locus interaction containing GAB2, rs10510511, and rs197899; and discovers as its second highest scoring model, a 3-locus interaction containing GAB2, SPAG16, and APOE. The MDL score also discovers this latter interaction. The interaction strengths (*IS*) of these two models is much greater than the *IS* of the 2-SNP models. So, our results support that there might be other loci involved in the GAB2/APOE interaction. We know of no previous research indicating this. Furthermore, there is previous research indicating APOE and the SPAG16 gene interact to affect LOAD, but not with GAB2 [59].

Table 4 shows all the loci that appeared in learned interactions and their individual information gains. The third locus shown is the locus providing the most information gain other than APOE and APOC1. This locus does not appear in an interaction. We include it to show that, other than APOE and APOC1, no locus provides substantial information gain by itself. On the other hand several of the interactions provide substantially more information gain that APOE or APOC1 do individually. For example, the fifth model in Table 2 includes APOE and has an *IS* of 0.049. Recall that the *IS* is the information gain provided by all the loci in the model taken together minus the sum of their individual information gain. Since the information gain provided by APOE by itself is 0.121 (see Table 4), this fifth model provides better than a 0.049/0.121 = 0.40 increase in information gain over APOE by itself. The seventh model in Table 2 includes APOC1 and also has an IS of 0.49. Since the information gain provided by APOC1 by itself is 0.080 (see Table 4), this seventh model provides better than a 0.049/0.080 = 0.61 increase in information gain over APOC1 by itself.

**Table 4:** The loci involved in the 14 interactions learned using the BDeu score or the 21 interactions learned using the MDL Scored. The second column shows their rank when we score all 312,260 1-SNP models using the MDL score. The third column shows the information gain provided by the SNP by itself. The SNP in the third row is not in a learned interaction. It is included to show the highest scoring SNP other than APOE or APOC1.

Locus	Rank	Info Gain
APOE	1	0.121
rs4420638 (APOC1)	2	0.080
rs6784615	3	0.016
rs1785928	111	0.010
rs41528844	449	0.008
rs7355646	968	0.007
rs1389421	966	0.007
rs8108841	994	0.007
rs11895074	1085	0.007
rs7115850	1384	0.006
rs891159	1599	0.006
rs41369150	1781	0.006
rs898717	2731	0.006
rs17095891	2817	0.006
rs10510511	2901	0.006
rs16975605	3404	0.005
rs197899	3915	0.005
rs6719419	4219	0.005
rs536128	4841	0.005
rs2057537	4813	0.005
rs1957731	5781	0.005
rs6121360	6887	0.005
rs10130967	6901	0.005
rs986647	7820	0.005
rs10793294	7983	0.004
rs10922885	8045	0.004
rs2717389	8314	0.004
rs41491045	8527	0.004
rs12421071	8736	0.004
rs12674799	8741	0.004
rs11676052	8804	0.004
rs7101429	11208	0.004
rs11122116	13898	0.004
rs16992170	18747	0.004
rs11217838	20371	0.004
rs2265264	99260	0.001
rs16856748	108717	0.001
rs734600	138078	0.001

Table 5 lists the genes that appeared in learned interactions, and shows whether previous research indicated associations of those genes with LOAD. We see that many of our findings are new; however, we have also substantiated previous research.

**Table 5:** The genes involved in the 14 interactions learned using the BDeu score or the 21 interactions learned using the MDL Scored. The second column whether previous research indicated whether they were involved in an interaction concerning LOAD, and the third column shows whether previous research indicated that they had an effect on LOAD when interactions were not considered.

Gene	Prev. Int. Effect	Previous No Int. Effect
APOE	Yes	Yes
APOC1	Yes	Yes
GAB2	Yes	No
SPAG16	Yes	No
CALN1	No	No
NPHP4	No	No
LRP2	No	Yes
EYA2	No	No
FNDC3B	No	No
ARHGEF12	No	No
ADAMTS16	No	Yes
TM9SF4	No	No
ELP2	No	Yes
TCP11	No	No
ATG10	Yes	No
ZNF77	No	No
FRMD4A	No	Yes

S2 Table shows the running times in the various analyses performed in the LOAD study. These results seem to contradict those in S1 Table. That is, in the real analysis the MDL score seems more efficient that the BDeu score. The explanation would be that in the real study the MDL score usual ends its search much sooner due to its larger DAG penalty. Tables 2 and 3 show that the MDL score does learn smaller models.

## Discussion

We presented MBS-IGain, a method for identifying interactive effects in high-dimensional datasets. Based on our experiments, MBS-IGain is highly effective at doing this, substantially exceeding other methods. The effectiveness of MBS-IGain is owing to its three components. First, if possible MBS-IGain initiates a beam from every predictor because we have no way of determining if it is involved in an interaction without investigating its effect in combination with other predictors. We noted that MBS-IGain should be able to handle 20,000 predictors in about 12 days. Nevertheless, in studies such as GWAS we often have millions of predictors. In these cases, we must prune the number down to a manageable size. In our investigation we did this by scoring all one-predictor models, and choosing the ones with the best score. Other strategies, such as the use of ReliefF [13] could be employed. Second, MBS-IGain uses information gain to determine whether to add a predictor on a given beam rather than using the score. In this way, we identify predictors that are interacting rather than merely identifying high scoring models. Third, MBS-IGain uses the score to end its search on each beam. In this, we only identify interactions that are likely models. These three components together are essential to the performance of MBS-IGain.

We applied MBS-IGain to a real LOAD dataset. We obtained new results, most notable are results indicating that there are more loci involved in the APOE/GAB2 interaction; and we also substantiated previous findings. These results not only substantiate the efficacy of MBS-IGain, but also provide LOAD researchers with avenues for further investigation.

In related research, Hu et al. [60] used information gain to link interacting SNPs in statistical epistasis networks. However, their technique was limited to investigating two-SNP interactions for the purpose of constructing a network. It did not search multiple beams, investigate higher-order interactions, or use the Bayesian score to judge whether an apparent interaction was a likely model.

## Supporting Information

S1 Dataset Simulated datasets used in the experiments. (ZIP)

S1 Fig *Power*_1_(*K*) for MBS-IGain using the MDL score with *T* = 0.1 and the BDeu score with α = 4 and *T* = 0.1. (TIF)

S2 Fig *Power*_2_(*K*,*C*) for MBS-IGain using the MDL score with *T* = 0.1 and the BDeu score with α = 4 and *T* = 0.1. (TIF)

S1 Table Average running times to process the 100 1000 SNP simulated datasets. (DOCX)

S2 Table Running times for the LOAD study. (DOCX)
