# Supplementary figures and images for: Learning Predictive Interactions Using Information Gain and Bayesian Network Scoring
Source: PLoS One. 2015 Dec 1;10(12):e0143247. doi: 10.1371/journal.pone.0143247 (PMC4666609; doi:10.1371/journal.pone.0143247)

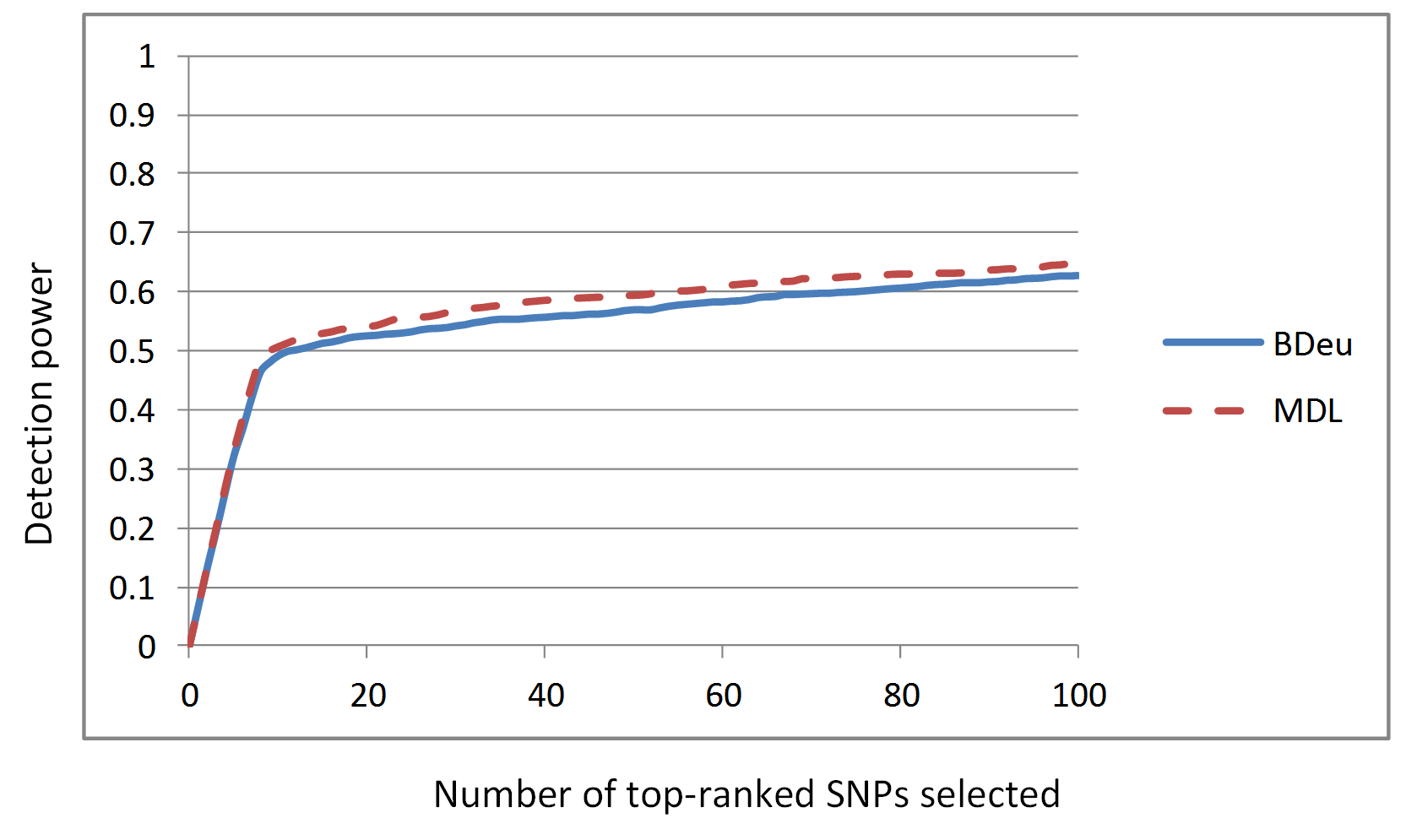

Supplement: S1 Fig — (TIF) [file pone.0143247.s002.tif]

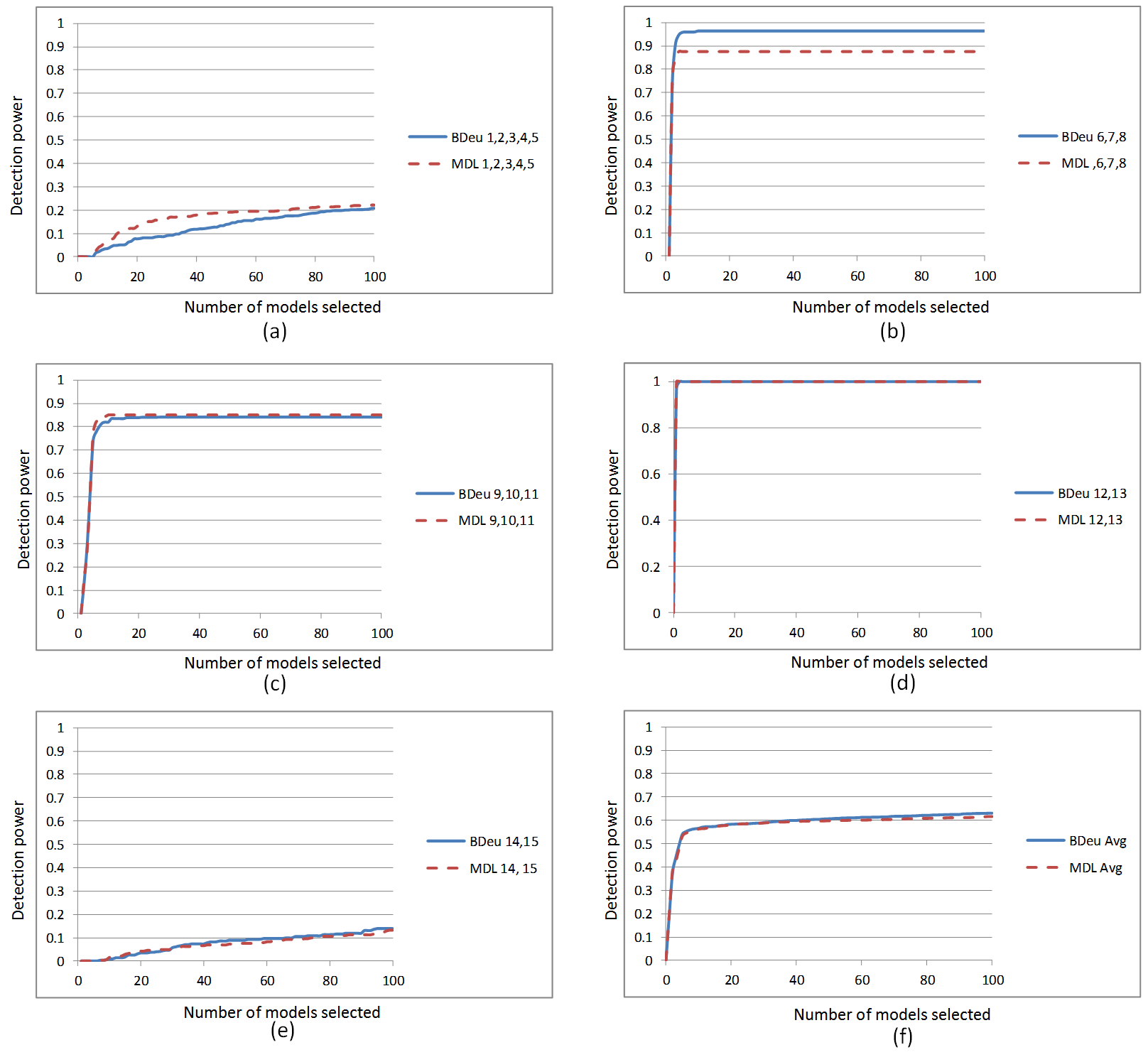

Supplement: S2 Fig — (TIF) [file pone.0143247.s003.tif]
