# Supplementary material for: Learning Predictive Interactions Using Information Gain and Bayesian Network Scoring
Source: PLoS One. 2015 Dec 1;10(12):e0143247. doi: 10.1371/journal.pone.0143247 (PMC4666609; doi:10.1371/journal.pone.0143247)
Supplement: S1 Table — (DOCX) [file pone.0143247.s004.docx]

**S1 Table.** Average running times to process the 100 1000 SNP simulated datasets.

| **Score** | **MBS-IGain** | **Regal** | **MBS** |
| --- | --- | --- | --- |
| BDeu | 26.68 min | 136.93 min | 46.20 min |
| MDL | 45.20 min | 237.72 min | 77.50 min |
