# Supplementary material for: Learning Predictive Interactions Using Information Gain and Bayesian Network Scoring
Source: PLoS One. 2015 Dec 1;10(12):e0143247. doi: 10.1371/journal.pone.0143247 (PMC4666609; doi:10.1371/journal.pone.0143247)
Supplement: S2 Table — (DOCX) [file pone.0143247.s005.docx]

**S2 Table.** Running times for the LOAD study.

| **Score** | **1000 SNPs** | **5000 SNPs** | **10,000 SNP** | **100 × 312,260 SNPs** |
| --- | --- | --- | --- | --- |
| BDeu | 57.99 min | 27.88 hours | 4.71 days | 27.37 hours |
| MDL | 23.11 min | 9.26 hours | 1.36 days | 5.60 hours |
